# Investigate the Efficacy of Dual-Target Electrical Stimulation in the Treatment of Knee Osteoarthritis After Stroke and its Effect on Cerebral Cortical Activity: A Randomized Controlled Trial

**DOI:** 10.1155/np/2886215

**Published:** 2025-08-06

**Authors:** Chun-Ya Xia, Hui-Fang Tian, Xu-Yan Ren, Zhi-Hang Xiao, Hui-An Chen, Yi-Jia Yin, Le-Chi Zhang, Si-Yan Cai, Ting-Ting Li, Jun Zou, Jie Bao, Min Su

**Affiliations:** ^1^Department of Rehabilitation Medicine, The Fourth Affiliated Hospital of Soochow University (Suzhou Dushu Lake Hospital), Suzhou, Jiangsu, China; ^2^Institute of Rehabilitation, Soochow University, Suzhou, Jiangsu, China; ^3^Department of School of Mathematics and Physics, Xi'an Jiaotong-Liverpool University, Suzhou, Jiangsu, China; ^4^Department of Orthopedic Surgery, The First Affiliated Hospital of Soochow University, Suzhou, Jiangsu, China; ^5^Department of School of Physical Education and Sports Science, Soochow University, Suzhou, Jiangsu, China

**Keywords:** chronic pain, knee osteoarthritis, stroke, transcranial direct current stimulation, transcutaneous electrical nerve stimulation

## Abstract

Transcranial direct current stimulation (tDCS) and transcutaneous electrical nerve stimulation (TENS) are both recognized for their analgesic effects; however, evidence suggests limitations in their efficacy when applied to knee osteoarthritis (KOA) after stroke. This study aimed to assess the efficacy and cortical activity impact of a dual-target electrical stimulation approach combining tDCS and TENS in the treatment of KOA after stroke. We hypothesized that the combination of tDCS with TENS could more effectively address KOA after stroke by enhancing brain activity through the induction of neural oscillations. To test this hypothesis, a double-blind, randomized trial was conducted with 30 participants receiving either TENS + tDCS or TENS + sham tDCS over an 8-week period, from Monday to Friday. Electroencephalograms (EEGs), Brief Pain Inventory (BPI), visual analog scale (VAS), stride length, cadence, 6-min walk test (6 MWT), knee range of motion (ROM), and quadriceps strength were collected pre- and poststimulation. Pain indicators were analyzed using *t*-tests for continuous variables and chi-square tests for categorical variables, with repeated measures ANOVA employed to explore changes and interactions over time. For EEG analysis, paired *t*-tests were utilized to investigate changes in brain regions before and after treatment on the affected side, with visual analysis conducted subsequently. The results indicated that the combined treatment led to significant improvements in the affected hemisphere, with significant changes observed in α1, α2, and β power. Additionally, significant group× time interaction effects were noted for BPI, VAS, stride length, cadence, and 6MWT. The study concludes that dual-target electrostimulation using tDCS combined with TENS significantly ameliorates knee joint inflammation following stroke by acting on the cerebral cortex and target organs.

**Trial Registration:** Chinese Clinical Trial Registry: ChiCTR2200064735

## 1. Introduction

Stroke is the second leading cause of mortality globally [1], about 50%–80% of them will be left with some degree of disability [2]. Lower limb motor dysfunction is a prevalent poststroke complication, affecting over half of stroke survivors [3]. Due to neurological deficits and alterations in connective tissue, stroke patients exhibit reduced muscle strength and decreased joint mobility on the affected side [4, 5], predisposing the knee joint to injury. Furthermore, as stroke patients generally aim to regain ambulatory capacity [6], they are more inclined to consciously exercise their affected lower limbs during daily activities. This process, due to the inability to accurately maintain balance before training and rest, can further lead to wear and tear on the knee cartilage, which are risk factors for knee osteoarthritis (KOA) [7].

KOA is a chronic degenerative joint disease precipitated by various factors, including age, metabolic bone disease, obesity, and acute or chronic joint injuries [8]. Its characteristics include pathological changes in cartilage, bone, synovium, ligaments, muscles, and fat around the joint, leading to joint dysfunction, pain, stiffness, functional limitation, and loss of valuable activities [9]. With the rising prevalence of obesity and an aging population, the incidence of KOA is increasing [10]. However, there is currently no definitive cure for KOA, and alleviating patient pain remains the primary strategy for its treatment [11, 12].

Transcutaneous electrical nerve stimulation (TENS) is a form of peripheral nerve stimulation frequently applied in various clinical settings to alleviate pain, offering an alternative to pharmacological interventions [13]. Animal studies have demonstrated that TENS activates endogenous inhibitory mechanisms to reduce central excitability [14]. TENS controls pain by delivering electrical currents through the skin, with its pain improvement mechanisms grounded in the “gatekeeper theory” of pain modulation, which is one of the theories explaining the inhibition of pain signals [15]. According to this theory, TENS inhibits the transmission of nociceptive signals from small-diameter fibers (A-delta and C) by activating inhibitory interneurons in the gelatinous substance of the dorsal horn of the spinal cord (SDH) through electrical stimulation of large-diameter fibers (A-β fibers). However, due to the uncertainty of TENS's clinical efficacy and limited high-quality evidence, its effectiveness remains controversial [16]. The National Institute for Health and Clinical Excellence (NICE) does not recommend the use of TENS for labor care or nonspecific chronic low back pain but recommends its use as an adjunctive treatment for osteoarthritis and rheumatoid arthritis [17]. Given that TENS is a portable, safe, and low-cost treatment method, it is necessary to explore the efficacy of combining TENS with another form of noninvasive stimulation.

The application of anodal transcranial direct current stimulation (tDCS) in the M1 area has shown positive effects on pain relief [18, 19]. It generally involves applying weak direct current to the scalp using sponge electrodes. It has been proven that tDCS can influence the excitability of cortical regions below the electrode and distant areas connected to the primary stimulation zone, with effects lasting for several hours [20]. Anodal tDCS in the primary motor cortex regulates pain through direct cortical effects on the ventral nucleus of the thalamus and anterior nucleus, as well as downstream effects on the medial thalamus, prefrontal cortex, and upper brainstem. Preliminary clinical trials have indicated that anodal tDCS in the primary motor cortex may be effective in treating chronic pain conditions, such as SDH injury, fibromyalgia, and chronic pelvic pain [21]. However, studies using finite element model (FEM) computational modeling suggest that due to the low focalization and dispersion of tDCS current density as it passes through the scalp, this may result in less actual efficacy in clinical applications. The use of tDCS alone without any other combined interventions may be one reason for its low efficacy [22].

In addition to using pain improvement as an indicator of KOA relief, we also plan to analyze cortical electrical activity associated with tDCS stimulation, a research approach that has not yet been applied clinically. Studying the electrophysiological measurements induced by tDCS therapy may help us better understand its mechanism of action. For this purpose, electroencephalography stands out as a tool for monitoring therapeutic responses [23, 24].

Both tDCS and TENS treatments can alleviate pain, but both methods have certain limitations when treating KOA after stroke [25]. Considering that anodal tDCS can modulate central pain processing capabilities and increase the brain's receptivity to other interventions through an “onset” effect, adding anodal tDCS to TENS may complement pain mechanisms and enhance the brain's responsiveness to motor analgesia, thereby producing more positive clinical outcomes in KOA [26]. We propose a dual-target electrical stimulation approach combining tDCS and TENS, hypothesizing that tDCS combined with TENS can improve brain activity by modulating α and β rhythms. To this end, we designed a double-blind, randomized trial with 30 participants in each group receiving TENS + tDCS and TENS + sham tDCS. We anticipate that the combined intervention may be more effective for KOA after stroke compared to TENS alone.

## 2. Methods

### 2.1. Subjects

**Figure 1:**
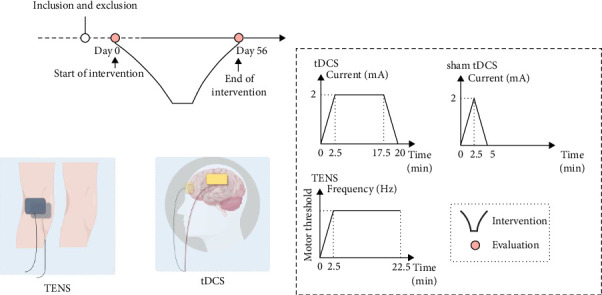
The study timeline.

This study has been approved by the local ethics review committee and was conducted in accordance with the World Medical Association's Code of Ethics (Declaration of Helsinki) for experiments involving human subjects. The study procedures were explained to all participants before their involvement, and a written informed consent form was provided for all participants to participate in the study and to publish the results. The study schedule is shown in Figure 1.

The figure give a timeline of the study, a schematic of the treatment of transcutaneous nerve electrical stimulation and tDCS, including electrode placement and treatment parameters.

Participants were recruited in Suzhou from May 2021 to May 2022. Inclusive criteria were as follows: (1) a history of stroke for at least 1 year; (2) muscle strength of the key muscles of the lower limbs is greater than or equal to level 4; (3) MRI-confirmed KOA; (4) no history of KOA prior to stroke; (5) participants aged between 18 and 80; and (6) visual analog scale (VAS) between 3 and 8.

Exclusion criteria were as follows: (1) undergoing physiotherapy or surgery; (2) pain in other parts of the body that was more severe than the knee, causing significant physical activity impairment; (3) use of life-sustaining medication; (4) failure to sign the informed consent form or being considered ineligible for this study; (5) presence of contraindications to the treatment, such as having a history of epilepsy, severe cognitive and communication disorders that do not cooperate with evaluation and treatment, wears a pacemaker, have metal implants in the skull, or have skull defects, complete internal carotid artery occlusion, direct damage to the stimulation area, vestibular dysfunction, pregnant women, etc.; (6) severe pain and inability to discontinue analgesics.

### 2.2. Sample Size Calculation, Random Assignment, and Blinding

In this randomized controlled trial, the experimental group received TENS + tDCS, while the control group received TENS + sham tDCS.

To minimize confounding effects, participants were instructed to refrain from using analgesics (including NSAIDs and opioids) throughout the trial. Adherence to this protocol was monitored through: Daily self-reporting: Participants maintained standardized logs documenting any medication use. Weekly verification: Research staff conducted structured telephone interviews to crossvalidate self-reported data. Pharmacy record review: Prescription databases were screened to confirm the absence of analgesic prescriptions during the study period.

We used R 4.2.2 for calculations with a power of 90% and a significance level of 0.05, obtaining a sample size of 30 participants for each group. Considering a 7% dropout rate, the total sample size was 65 cases.

The 65 participants were randomized to either the experimental group (TENS + tDCS) or the control group (TENS + sham tDCS) using a computer-generated block randomization method (block size = 6, with a random seed). The randomization and allocation concealment procedures were implemented as follows: Random sequence generation: An independent statistician generated the randomization sequence using SAS 9.4 software, with four randomization blocks (block size = 6). The final block allowed incomplete filling to accommodate the total sample size of 65. Allocation concealment: A third-party research coordinator, uninvolved in the trial, sealed each participant's unique identification number (1–65) and corresponding group assignment in sequentially numbered, opaque envelopes.

The envelopes were secured with tamper-evident seals (e.g., stamped overlapping edges) and opened by the coordinator under video surveillance only after baseline assessments were completed. Blinding safeguards: Researchers, participants, and outcome assessors were blinded to the randomization sequence and envelope management system. Group assignments were unblinded solely during the data analysis phase.Therapists operating TENS/tDCS also do not know the grouping.

### 2.3. tDCS Protocol

Both groups of patients will receive an 8-week intervention from Monday to Friday. The TENS and tDCS interventions are administered by physical therapists who were not involved in the study design and data analysis. They will document and monitor participant adherence and ensure timely communication with participants to avoid missing treatment. All therapists completed a 16-h training program covering: device parameterization; anatomical landmarks for electrode placement; emergency protocols.

Five participants withdrew from the study before the experiment. Among them, four reported mild discomfort on their scalp (two in the experimental group and two in the control group), and one participant in the control group experienced a slight headache during the tDCS procedure, the other subjects did not experience discomfort. The therapists documented these situations and stopped treatment for these five participants according to the protocol, followed up with their conditions subsequently. The discomfort symptoms were mild and resolved quickly, not affecting their regular activities. The remaining 60 participants did not report any adverse reactions related to the intervention during the treatment process.

#### 2.3.1. TENS

The TENS intervention involved placing two electrodes on the patella of the affected knee and setting the TENS parameters as follows: scan mode in the 1–250 Hz range, symmetrical bipolar pulses, 60 ms pulse width [27]. The intensity will be gradually increased, and the stimulation duration will be 20 min per session. Electrode placement location: Medial; placed in the medial space of the knee joint (about 2–3 cm below the patella, near the medial condyle of the tibia), Lateral; placed in the lateral space of the knee joint (in front of the fibula).

#### 2.3.2. tDCS

The tDCS delivers current through saltwater-soaked surface sponge electrodes (5 × 7 cm). At the beginning of the intervention, the current increases from 0 to 2 mA within 2.5 min. After 15 min of 2 mA stimulation, the current decreases from 2 to 0 mA within 2.5 min. The anode is placed on the C3 area of the affected side of the brain, corresponding to the primary motor cortex (M1), while the cathode is located in the supraorbital area (Figure 1) on the other side. The sham stimulation protocol for the control group is identical to that of the experimental group, experiencing the same process of current increase and decrease, but without continuous 2 mA stimulation. For the sham tDCS group, After the current increases from 0 to 2 mA in 2.5 min, and then decreases to 0 mA in 2.5 min, while the electrodes remained in position with an active beeping sound. At the end of each treatment, participants will be asked about any adverse reactions to ensure the safety of the experimental protocol.

### 2.4. Evaluation

The primary observation indicators for this trial were electroencephalogram (EEG), Brief Pain Inventory (BPI). Secondary outcomes included the VAS, stride length, rhythm, 6-min walk test (6 MWT), range of motion (ROM) of the knee joint, and quadriceps strength. These indicators were assessed on the day before treatment and the day after discontinuation of treatment.

#### 2.4.1. EEG

In this experiment, EEG data of 30 individuals in the experimental group were collected before and after 10 min of rest, and the data were compared and analyzed. The BCI-Assessment wireless multichannel EEG acquisition and analysis system (manufactured by Xian Zhen Tai Company) was used. To maintain the position of EEG electrodes (Fp1, Fp2, Fz, F3, F4, F7, F8, Fcz, Fc3, Fc4, Ft7, Ft8, Cz, C3, C4, T3, T4, CPz, CP3, TP7, TP8, CP4, Pz, P3, P4, T5, T6, Oz, O1, and O2) on the scalp during different measurement periods, the length from the root of the nose to the external occipital protuberance and the distance from the right to the left ear point were recorded for all participants. Throughout the data collection process, impedance was checked and kept below 5 kΩ. The average value of the bilateral mastoid region (M1 + M2)/2 was used as the recording reference. The low-pass filter was set to 0.03 Hz, the high-pass filter was set to 60 Hz, and the notch filter was set to 50 Hz to reduce power line artifacts. The raw EEG data were recorded at a sampling rate of 1000 Hz, and the analog-to-digital converter was set to 500 Hz.

The extracted EEG data to be analyzed was preprocessed using MATLAB 2021a and EEGLAB toolbox.The power spectral density (PSD) of brain wave data has been plotted. Power spectral analysis involves analyzing the frequency components and power distribution of signals through the Fourier transform. The calculation of the power spectrum involves calculating the square amplitude values of each frequency component, then taking the logarithm of the power, and converting the result to decibels (dB) to enhance visualization. The power spectrum can reveal the energy distribution of brain signals at different frequencies, aiding in analyzing the intensity of certain brain wave activities. When plotting the spectrum, the *x*-axis represents frequency, and the *y*-axis represents power. Draw relative power topographic maps. Relative power analysis is a widely used quantitative method in EEG data processing for evaluating the relative contribution of EEG activity within specific frequency ranges. This method estimates the absolute power of each frequency band through PSD and normalizes the absolute power of a specific frequency band as a proportion of total power to calculate its relative power. Relative power values are mapped to each electrode position, and a two-dimensional distribution map of the entire scalp is generated using interpolation methods (bilinear interpolation or radial basis function interpolation). Since we only studied the affected side, we averaged the data from the left affected side (16 cases) and the right affected side (14 cases), separately drawing two topographic maps and combining the affected hemisphere images to observe changes in each region before and after treatment. The topographic maps were generated using EEGLAB by invoking the drawing function to visually display the spatial distribution characteristics of specific frequency bands.

#### 2.4.2. BPI

BPI was collected from participants prior to the motor ability and mechanics assessment and used to assess the intensity of the subjects' pain over the past 24 h, quantifying the pain scale from 0 (painless) to 10 (the most severe pain). A score of 3 or less means mild, bearable pain; a score of 4–6 means that the pain interferes with sleep but is bearable; and a score of 7–10 means that the participant suffers from progressively more intense, unbearable pain that interferes with appetite and sleep. This study collected four indicators of the patients' pain over the past 24 h: most intense, least intense, average, and current pain, and averaged them to analyze the pain level.

#### 2.4.3. VAS

Participants' VAS were collected after motor ability and mechanical assessments. The VAS was utilized to help the participant determine the average level of pain in the past 2 weeks, where the 100-mm scale displays the numbers “0 (painless)” and “10 (the most severe pain)”.

#### 2.4.4. Gait Analysis (Step Length and Cadence) and 6 MWT

The Gaitboter gait analysis system (Zhongke Huicheng Technology, Beijing, China) is used for gait collection and analysis. This system consists of wearable gait acquisition equipment and analysis software. The parameters of the subjects' step length and cadence were recorded at the end of the test. During the test, the participant walked 20 m back and forth on the ground once. Before starting the test, the test procedure and precautions were explained to the subjects to ensure their complete understanding and cooperation. The parameters of the subjects' step length and cadence were recorded at the end of the test.

The 6 MWT tests a patient's ability to walk continuously over a 6-min walking distance. Before starting the test, participants need to rest enough to be in good condition to complete the test. The test was conducted on a flat, hard surface over 50 m. During the first 6 min of the test, participants were not allowed to wear watches or other objects and were encouraged to walk as far as possible. Participants were allowed to rest during the test, but the rest period was included in the test time. When the test time was over, the distance traveled by the participants was recorded.

#### 2.4.5. Active ROM and Quadriceps Strength

In this study, the participant was placed in a supine position, and the position of the knee joint was measured with a protractor from the maximum extension pull to the maximum flexion position.

We used the hand-held dynamometer (HHD) (TB5-NK-300 N, HAIFUDA Technology, Beijing, China) to assess the maximum isometric voluntary contraction force of the quadriceps muscle. Participants were asked to sit upright while the knee joint was in 90° flexion. The dynamometer was placed 2 in. near the lateral portal bone of the anterior tibia, and the leg was stabilized with a nonelastic band. This strap was designed to hold the ergometer on the front of the leg and was secured to the base of the table.

### 2.5. Statistical Analysis

This study used the Social Science Statistical Software Package (SPSS) version 27.0.1 for statistical analysis applicable to the Windows system. *T*-tests were used to compare numerical variables at baseline, while chi-square tests were used to compare categorical variables. All baseline indicators were assessed in both groups before treatment initiation (T0). There was no statistically significant difference between the two groups (Table 1). *p*-Values less than 0.05 were considered statistically significant differences.

In terms of inferential analysis, through the Shapiro–Wilk test (due to the small sample size, the results of the Shapiro–Wilk normality test are directly observed), the data of each group follows a normal distribution *p*  > 0.05. Since this experiment consists of only two groups-comparisons before and after two time points there is no way to compare the variances of differences between repeated measurements because there is only one variance, thus no sphericity test exists. A two-way repeated measures ANOVA method is adopted to evaluate the efficacy of wearable TENS combined with tDCS in treating KOA after stroke. Subsequently, Bonferroni correction is used to analyze the interaction effect.

For the EEG data, we averaged the raw absolute power of each frequency band (δ, θ, α1, α2, β1, and β2) on the corresponding electrodes in the frontal, M1, parietal, and occipital regions of the affected side. These data were used for statistical analysis. First, we performed a Shapiro–Wilk test to check the normality of the data, with all groups showing a normal distribution *p*  > 0.05. Then, we conducted a *t*-test for statistical analysis to investigate the changes in brain regions during TENS combined with tDCS treatment for KOA after stroke.

## 3. Results

**Figure 2:**
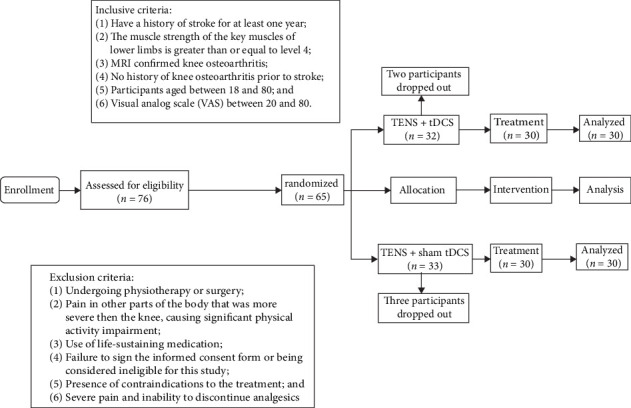
The CONSORT flow diagram.

Participants who did not complete the intervention and evaluation were not subjected to statistical analysis. Five participants withdrew from the trial before T1 due to mild discomfort, while 60 participants (30 in each group) received full treatment. The CONSORT flowchart is as follows: (Figure 2).

### 3.1. BPI

Repeated measures ANOVA results show: the main effect of group is not significant (*F* = 0.45, *p*=0.5, *η*^2^ = 0.008); the main effect of time is significant (*F* = 615.24, *p*  < 0.001, *η*^2^ = 0.914); the interaction effect of group × time before and after treatment is significant (*F* = 14.83, *p*  < 0.001, *η*^2^ = 0.204). Using Bonferroni correction method to analyze interaction effects, the simple effect test of time measurement results show that in group control, the simple effect of measurement times is significant (*F* = 219.52, *p*  < 0.001, *η*^2^ = 0.791); in group experimental, the simple effect of measurement times is significant (*F* = 410.55, *p*  < 0.001, *η*^2^ = 0.876). The simple effect test of group results show: before the experiment, the simple effect of groups is not significant, *F* = 2.48, *p*=0.12, *η*^2^ = 0.04; after the experiment, the simple effect of groups is significant, *F* = 20.49, *p*  < 0.001, *η*^2^ = 0.261. Multiple comparisons find that at T0, the mean difference between group experimental and group control is 0.367, with a significance level of 0.121, indicating no significant difference. At T1, the difference between group experimental and group control is −0.558, with a significance level less than 0.001, indicating a significant difference.

### 3.2. Other Indicators

In the VAS pain assessment, the main effect of group was not significant (*F* = 3.09, *p*=0.084, *η*^2^ = 0.051); the main effect of time was significant (*F* = 713.58, *p*  < 0.001, *η*^2^ = 0.93); and the interaction effect of group × time was also observed to be significant before and after treatment (*F* = 9.20, *p*=0.004, *η*^2^ = 0.137).

When analyzing interaction effects, the measurement time simple effect test results show that in group control, the simple effect of time is significant (*F* = 280.36, *p*  < 0.001, *η*^2^ = 0.829); in group experimental, the simple effect of time is also significant (*F* = 442.42, *p*  < 0.001, *η*^2^ = 0.884).

Group experimental showed better efficacy than group control, with statistically significant differences in VAS scores at posttreatment (T1) between the two groups (*p*  < 0.001, *F* = 16.78, *η*^2^ = 0.224, mean difference = 6.00). There was no significant difference at T0 (*F* = 0.17, *p*=0.678, *η*^2^ = 0.003).

The interaction effect of group × time was observed in the walking ability evaluation of step length and 6 MWT (*F* = 17.60 and *F* = 35.37, *p*  < 0.001 and *p*  < 0.001, *η*^22^ = 0.233, and *η*^22^ = 0.379), with the experimental group showing better efficacy than the control group. There were statistically significant differences in the two walking ability scores between the two groups at posttreatment (T1) (*p*  < 0.001 and *p*  < 0.001, *F* = 26.06 and *F* = 24.04, *η*^22^ = 0.310, and *η*^22^ = 0.293, mean difference values of − 6.73 and − 34.41). There was no significant difference at T0 (*F* = 2.48 and *F* = 0.02, *p*=0.121 and *p*=0.888, *η*^22^ = 0.041, and *η*^22^ < 0.000).

In the two groups, a significant time × group interaction was also observed in cadence (*F* = 14.67, *p*  < 0.001, and *η*^2^ = 0.202), and there was no significant difference in cadence scores before and after treatment (*p*=0.532 and *p*=0.06, *F* = 0.41 and *F* = 3.67, *η*^2^ = 0.007 and *η*^2^ = 0.059, mean difference of 0.87 and −2.60).

There was no significant time × group interaction in ROM (*F* = 3.68, *p*=0.06, and *η*^2^ = 0.06) and quadriceps strength (*F* = 1.12, *p*=0.294, and *η*^2^ = 0.019). (Tables 2 and 3).

### 3.3. EEG

Through paired samples *t*-tests, we found that after TENS combined with tDCS treatment, overall, the α1 (*p*=0.041, *p*  < 0.001, *p*  < 0.001, *p*  < 0.001), α2 (*p*=0.004, *p*=0.006, *p*  < 0.001, *p*  < 0.015), β1 (*p*=0.004, *p*=0.008, *p*=0.039, *p*=0.001) frequencies in the affected sides frontal lobe, M1, parietal lobe, and occipital lobe four regions were significantly increased. For the β2 frequency band, except for the affected sides M1 region (*p*=0.049), there was no significant increase in other regions. Additionally, we found that the *θ* frequency band of frontal lobe (*p*=0.002) and occipital lobe (*p*=0.016) showed a significant decrease, while the Gamma frequency band showed a significant increase (*p*=0.035, *p*=0.036). There were no significant changes in these two frequency bands in the M1 and parietal lobe regions. For specific data, (Table 4).

**Figure 3:**
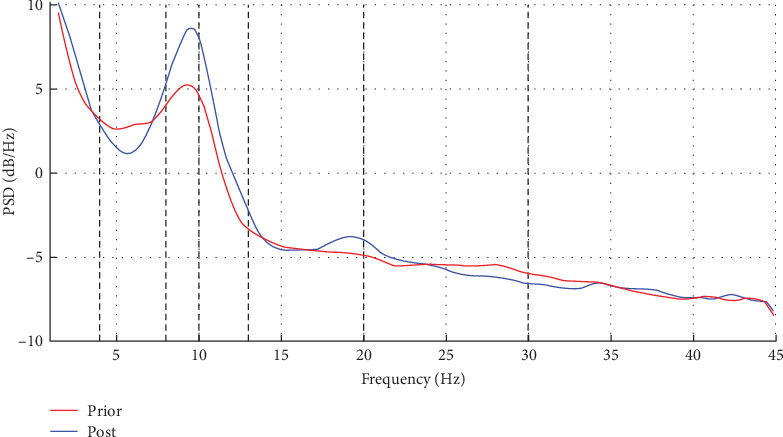
Brainwave data power spectrum diagram.

In addition to statistical analysis, we also conducted visual analysis: (Figure 3). After bilateral target electrical stimulation treatment, the power in α1 and α2 bands increased significantly, and the power in β band also increased.

The Figure shows the whole brain power spectrum of a representative experimental subject before and after receiving combination therapy.

**Figure 4:**
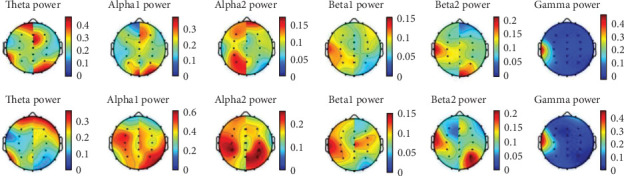
Relative power topographic map.

We separately plotted and overlaid the data from the left and right sides, creating a comparative Figure 4 of EEG relative power topography under six types of brainwave rhythms for TENS combined with tDCS treatment of KOA following stroke. From the topography, it can be seen that there is a significant increase in both the α and β1 frequency bands on the affected side.

The Figure was integrated with the EEG relative power topographic maps of the affected cerebral hemisphere of all the experimental subjects before and after treatment under six kinds of EEG rhythm.

**Figure 5:**
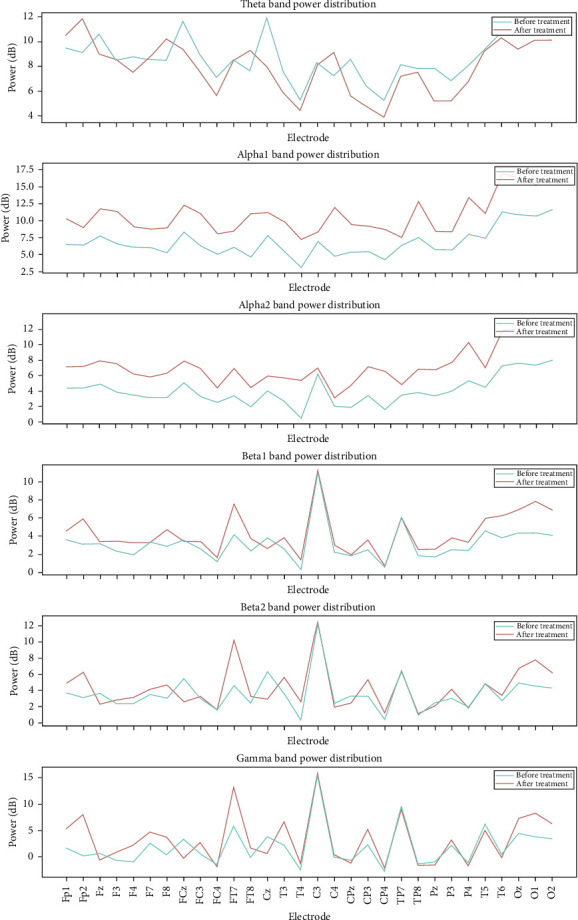
Changes of power distribution in different EEG frequency bands at different electrode positions.

Before and after treatment, the power distribution changes of different EEG frequency bands at various electrode positions were used to evaluate the effect of treatment on brain electrical activity (Figure 5). The results showed that the red curve was higher than the blue curve at most electrode positions after α1 and α2 treatment, indicating that the α band power related to relaxation state increased after treatment, and the brain may tend to be more relaxed. The frequencies of β1, β2, and gamma increased significantly after treatment in some frequency bands, especially FP1, FP2, FT7, O1, O2, and OZ. There was no obvious trend in θ wave.

This Figure shows the power distribution of different EEG frequency bands (θ, α1, α2, β1, β2, Gamma) before and after treatment at various electrode positions to evaluate the effect of treatment on brain electrical activity.

**Figure 6:**
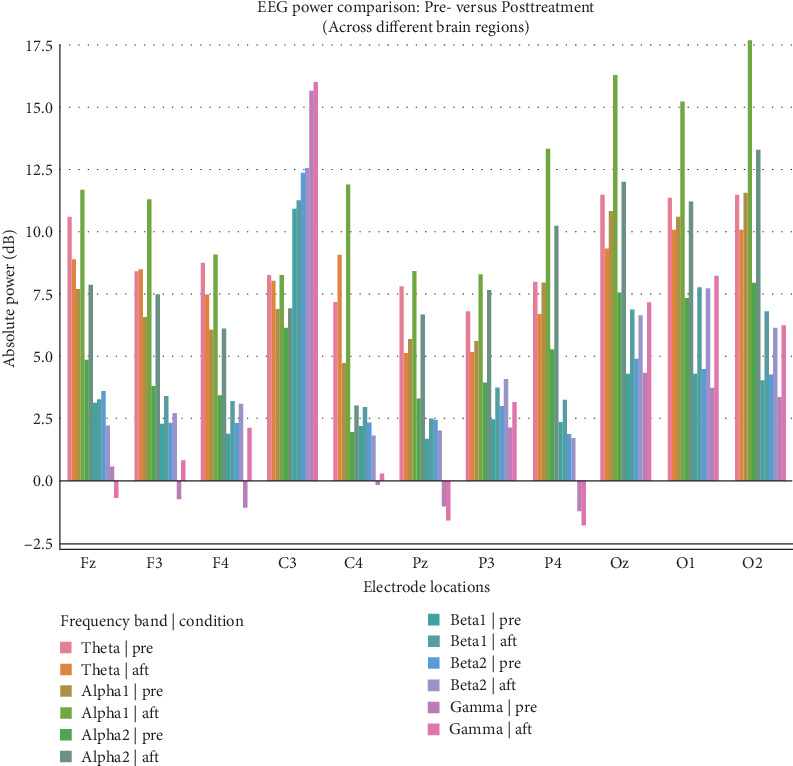
Bar graph of frequency band power comparison before and after treatment.

Comparison of EEG power at major electrode locations in M1 (C3, C4), frontal lobe (Fz, F3, F4), occipital lobe (O1, O2, Oz) and parietal lobe (Pz, P3, P4) in different frequency bands and conditions, data normalized based on baseline power (Figure 6). After treatment, the power of α1 and α2 waves increased significantly in 4 regions, especially in frontal lobe and occipital lobe. For Theta waves, the power of parietal lobe and occipital lobe decreased after treatment. The power of β1 and β2 waves increased slightly in each region before and after treatment, but only in occipital lobe. Gamma waves showed no trend except for occipital lobe.

This Figure shows a comparison of EEG power at major electrode locations in the M1 region (C3 and C4), frontal lobe (Fz, F3, and F4), occipital lobe (O1, O2, and Oz), and parietal lobe (Pz, P3, and P4) over different frequency bands and conditions, normalized to baseline power.

## 4. Discussion

This randomized controlled trial investigated the efficacy of TENS combined with tDCS in treating KOA after stroke and its impact on the cerebral cortex. The results showed that the combination therapy was effective, with all indicators showing significant improvement in the experimental group compared to the control group after 8 weeks. Notably, we found that the combination therapy alleviated KOA symptoms by affecting brain functional areas, particularly in pain control. Therefore, we hypothesized that dual-target electrical stimulation could improve KOA after stroke through both top-down and bottom-up approaches. tDCS primarily regulates pain and enhances the brain's receptivity to other interventions by affecting the M1 region of the brain in a top-down manner. Additionally, while TENS plays its role in pain control, it also gradually normalizes sensory receptors in the knee joint area, feeding back to the brain region in a bottom-up manner. The two approaches produce synergistic effects, enhancing analgesic efficacy. We also observed that dual-target electrical stimulation had significant effects on the frontal, parietal, and occipital lobes, but further research was not conducted on the specific regions affected.

This study demonstrates that the combined use of TENS and tDCS has a significant effect on treating KOA after stroke. After intervention, participants receiving TENS + tDCS treatment showed more pronounced improvements in pain reduction and walking ability. We not only evaluated the participants' pain levels but also their walking ability and biomechanical function of the knee joint. These results are similar to those obtained by Boggio et al. [28]. It is well known that chronic pain is not only associated with tissue damage but also with changes in higher central pain processing. The generation of KOA pain involves two pathways: one is the peripheral nociceptive pathway of the knee joint structure, and the other is the sensitization of both peripheral and central mechanisms. Persistent feedback from structural changes in the KOA joint increases synaptic excitability and efficiency in central pain pathways, leading to central sensitization characterized by localized and generalized hyperalgesia [29, 30], increased spinal excitability, and deficits in descending pain inhibition [31, 32]. Many patients with KOA have pain intensity associated with hyperalgesia and impaired descending pain inhibition [33]. TENS has the potential to modulate peripheral pain in the central nervous system [14]. And in cases of chronic pain, it may reduce the “pain” sensitivity of the central nervous system [34]. Anodal tDCS can modulate pain processing at the central level [35] and by adjusting the excitability of cortical neurons, it can increase the brain's receptivity to other interventions through an “initiation” effect [36]. Adding anodal tDCS to TENS may have a complementary effect on pain mechanisms and enhance the brain's responsiveness to motor analgesia, thereby producing more positive clinical outcomes in KOA.

The combination of tDCS and TENS can improve symptoms through a multitarget and multilevel mechanism of action. tDCS regulates the membrane potential of cortical neurons by applying low intensity direct current. Anodic stimulation can induce neuronal depolarization, activate volt-gated sodium channel and calcium channel, enhance cortical excitability, improve local metabolic environment by regulating ion channel (pannexin1 channel), promote the balance of electrical activity of neurons, and inhibit the diffusion of abnormal excitability in the brain injury area after stroke. TENS regulates the open state of sodium, potassium and calcium channels by activating Aβ fiber, and enhances the activity of sodium channels to inhibit pain signal transmission. tDCS and TENS regulate the function of ion channels from the central and peripheral, respectively, cooperate to restore the dynamic balance of neuronal excitability, and inhibit the abnormal transmission of central sensitization and peripheral pain signals after stroke

tDCS can inhibit the overexpression of BDNF/TrkB in the descending pain pathway after stroke. Experiments have shown that tDCS reduces central sensitization and reliefs chronic pain by reducing BDNF/TrkB protein levels in the ventromedial nucleus group (RVM) and dorsal horn of the SDH. tDCS regulates the activity of cortical glutaminergic neurons and GABAergic interneurons, reduces the excessive release of glutamate, enhances the transmission of inhibitory transmitters, and improves the hyperexcitability of neural networks after stroke. TENS can inhibit the release of local pro-inflammatory cytokines (such as IL-6, TNF-α), reduce the inflammatory response around the knee joint, and indirectly relieve pain. tDCS improves descending pain inhibition through central transmitter regulation, while TENS blocks pain signal upload through peripheral transmitter regulation, and the two work together to rebuild the “pain-inhibition” balance.

Alpha waves are associated with relaxation, meditation, creativity, and visualization processes. When people transition from a state of tension to relaxation, the amplitude of α waves increases. Beta waves are most pronounced when people are awake, active, thinking, or feeling stressed. They are related to higher cognitive functions of the brain, such as decision-making, language processing, and motor control. Our research has found significant differences in EEG indicators before and after treatment between participants in the combined therapy group: α waves and β1 waves in the M1 region of the brain were significantly enhanced, and EEG indicators in the frontal, occipital, and parietal lobes also showed some changes. This suggests that combined tDCS and TENS therapy may exert a more positive therapeutic effect by influencing brain function. This is consistent with previous reports. Li et al. [37]. have already found through research that TENS enhances the excitatory effects of tDCS on the PMC/SMA, and after tDCS + TENS intervention, activity in the unaffected hemisphere decreases, indicating that tDCS may balance activity in both hemispheres. This finding supports the view that tDCS can modulate target functional networks through neuroplasticity, suggesting that combined central and peripheral electrical stimulation may regulate pain by modulating the plasticity of specific functional neurons.

Poststroke central sensitization exacerbates pain perception in knee OA. Anodal tDCS over the primary motor cortex (M1) enhances GABAergic inhibition, downregulating hyperexcitability of wide dynamic range (WDR) neurons in the spinal dorsal horn, thereby disrupting the “pain-spasm-immobility” vicious cycle. TENS activates Aβ fibers to produce “gate-control” inhibition of *C*-fiber-mediated nociception, while its high-frequency stimulation (80–100 Hz) promotes endogenous opioid release—a critical mechanism in stroke patients with impaired endogenous analgesia. Motor function-joint loading feedback: Poststroke hemiparetic gait abnormalities accelerate cartilage degeneration. Combined therapy improves motor initiation efficiency via tDCS-mediated premotor cortex (PMC) modulation, while TENS reduces pain-related quadriceps inhibition, synergistically lowering peak joint stress. Long-term functional gains may decelerate OA progression through mechanical load redistribution.

Currently, tDCS or TENS acting on the nervous system of KOA patients at the neuromodulatory level is a promising therapeutic approach, although research in this area is limited, the use of [38, 39] alone or in combination with [28, 40] has shown limited efficacy. Therefore, we believe that this study will provide high-quality evidence that the combination of tDCS and TENS can improve the function of KOA patients after stroke by affecting brain functional areas. The results of the study will help refine clinical intervention decisions for chronic pain caused by KOA after stroke. Additionally, it can provide an efficient, noninvasive, and alternative treatment option for KOA patients after stroke, thus offering a crucial clinical conservative approach to this comorbid condition.

## 5. Limitation

This study has several limitations. First, this study did not design a pseudo TENS + tDCS group and a pseudo TENS + pseudo tDCS group. Future research should establish complete controls and explore the placebo effect during the intervention. Additionally, we mainly studied the changes in waveforms and their impact on pain in the affected side M1 area, but did not investigate the healthy side M1 area or further study the effects on the frontal, occipital, and parietal regions. All subjects were from the same region (Suzhou), which may limit the generalizability of the results, and future multicenter studies are needed to verify these results.

## 6. Conclusion

After 8 weeks of combined intervention, we observed that tDCS + TENS acted on the M1 region of the brain and knee joint to treat KOA after stroke, while promoting recovery in the corresponding brain regions through sensory feedback. In addition to the M1 region, the α1, α2, and β1 frequency bands on the affected side of the frontal lobe, occipital lobe, and cervical lobe were significantly increased.

## Figures and Tables

**Table 1:** Demographic and baseline characteristics.

Characteristics	Control group (*n* = 30)	Experimental group (*n* = 30)	*t*/*χ*^2^	*p*
Age, years, mean (SD)	63.70 (5.47)	65.31 (3.09)	−0.141	0.889
Female, *n* (%)	15 (50.00%)	15 (50.00%)	0.000	1.000
Male, *n* (%)	15 (50.00%)	15 (50.00%)	—	—
Kelgern–Lawrence grade I	23 (41.82%)	28 (50.91%)	0.914	0.339
Kelgern–Lawrence grade Ⅱ	32 (58.18%)	27 (49.09%)	—	—
Married	26 (86.67%)	28 (93.33%)	0.741	0.389
Unmarried	4 (13.33%)	2 (6.67%)	—	—
Retired	28 (93.33%)	29 (96.67%)	0.429	0.513
Unretired	2 (6.67%)	1 (3.33%)	—	—
Height, cm, mean (SD)	168.43 (8.28)	169.23 (8.34)	−0.373	0.711
Body weight, kg, mean (SD)	71.67 (8.90)	72.37 (9.04)	−0.302	0.764
BPI, mean (SD)	4.26 (0.73)	4.21 (0.70)	0.271	0.788
VAS, mean (SD)	49.33 (9.12)	50.23 (7.51)	−0.417	0.888
Step length, cm, mean (SD)	54.52 (1.77)	55.19 (1.54)	−1.576	0.121
Cadence, step·min^−1^, mean (SD)	89.00 (6.14)	88.13 (4.17)	0.642	0.524
6 MWT distance, m, mean (SD)	316.62 (24.80)	317.43 (19.65)	−0.141	0.888
ROM, degree, mean (SD)	87.03 (6.29)	86.84 (6.25)	0.114	0.910
Quadriceps strength, N, mean (SD)	24.11 (3.33)	24.33 (2.66)	−0.272	0.786

*Note:* Demographic characteristics of patients and each assessment measure at baseline in both groups. *p*  < 0.05 was considered significant.

Abbreviation: SD, standard deviation.

**Table 2:** All indexes during treatment and follow-up of the two groups mean and SD.

Measure	Group/Effect	T0	T1	Repeated measures *F*-test		
		Mean and SD	Mean and SD	*F*	*p*	Partial
BPI (score)	Control group	4.28 (0.76)	1.76 (0.21)	—	—	—
	Experimental group	4.64 (0.87)	1.2 (0.24)	—	—	—
	Group	—	—	0.45	0.5	0.008
	Time	—	—	615.24	<0.001*⁣*^*∗∗∗*^	0.914
	Group × time	—	—	14.83	<0.001*⁣*^*∗∗∗*^	0.204

VAS (mm)	Control group	49.33 (83.26)	22.40 (39.35)	—	—	—
	Experimental group	50.23 (56.39)	16.40 (26.62)	—	—	—
	Group	—	—	3.09	0.084	0.051
	Time	—	—	713.58	<0.001*⁣*^*∗∗∗*^	0.93
	Group × time	—	—	9.20	0.004*⁣*^*∗∗*^	0.137

Step length (cm)	Control group	54.52 (3.12)	59.01 (6.92)	—	—	—
	Experimental group	55.19 (2.36)	62.79 (9.56)	—	—	—
	Group	—	—	21.72	<0.001*⁣*^*∗∗∗*^	0.272
	Time	—	—	266.02	<0.001*⁣*^*∗∗∗*^	0.821
	Group × time	—	—	17.60	<0.001*⁣*^*∗∗∗*^	0.233

Cadence (step · min^−1^)	Control group	89.00 (37.66)	96.86 (36.88)	—	—	—
	Experimental group	88.13 (17.39)	99.46 (18.45)	—	—	—
	Group	—	—	0.46	0.501	0.008
	Time	—	—	448.53	<0.001*⁣*^*∗∗∗*^	0.885
	Group × time	—	—	14.67	<0.001*⁣*^*∗∗∗*^	0.202

6 MWT distance (m)	Control group	316.62 (615.13)	365.14 (725.56)	—	—	—
	Experimental group	317.43 (386.16)	399.54 (751.77)	—	—	—
	Group	—	—	9.31	0.003*⁣*^*∗∗*^	0.138
	Time	—	—	534.86	<0.001*⁣*^*∗∗∗*^	0.902
	Group × time	—	—	35.37	<0.001*⁣*^*∗∗∗*^	0.379

ROM (degree)	Control group	87.03 (39.55)	94.27 (66.44)	—	—	—
	Experimental group	86.84 (39.12)	96.89 (40.60)	—	—	—
	Group	—	—	0.58	0.449	0.01
	Time	—	—	139.53	<0.001*⁣*^*∗∗∗*^	0.706
	Group × time	—	—	3.68	0.06	0.06

Quadriceps strength	Control group	24.11 (11.07)	26.83 (14.02)	—	—	—
	Experimental group	24.33 (7.07)	26.14 (13.14)	—	—	—
	Group	—	—	0.10	0.750	0.002
	Time	—	—	27.90	<0.001*⁣*^*∗∗∗*^	0.325
	Group × time	—	—	1.12	0.294	0.019

Abbreviations: 6MWT, 6-min walking test; BPI, Brief Pain Inventory; ROM, range of motion; VAS, visual analog scale.

*⁣* ^*∗*^ *p*  < 0.05.

*⁣* ^*∗∗*^ *p*  < 0.01.

*⁣* ^*∗∗∗*^ *p*  < 0.001.

**Table 3:** Univariate tests and pairwise comparisons.

Measure	Time		Mean difference	*F*	*p*	Partial eta squared
BPI	T0	Contrast	—	2.475	0.121	0.041
	—	*I–J*	−0.367	—	—	—
	T1	Contrast	—	20.491	<0.001*⁣*^*∗*^	0.261
	—	*I–J*	0.558	—	—	—

VAS	T0	Contrast	—	0.174	0.678	0.003
	—	*I–J*	−0.900	—	—	—
	T1	Contrast	—	16.781	<0.001*⁣*^*∗*^	0.224
	—	*I–J*	6.000	—	—	—

Step length	T0	Contrast	—	2.483	0.121	0.041
		*I–J*	−0.673	—	—	—
	T1	Contrast	—	26.060	<0.001*⁣*^*∗*^	0.310
	—	*I–J*	−3.783	—	—	—

Cadence	T0	Contrast	—	0.412	0.523	0.007
	—	*I–J*	0.870	—	—	—
	T1	Contrast	—	3.665	0.060	0.059
	—	*I–J*	−2.600	—	—	—

6 MWT	T0	Contrast	—	0.020	0.888	0.000
	—	*I–J*	−0.817	—	—	—
	T1	Contrast	—	24.039	<0.001*⁣*^*∗*^	0.293
	—	*I–J*	−34.406	—	—	—

ROM	T0	Contrast	—	0.013	0.910	0.000
	—	*I–J*	0.184	—	—	—
	T1	Contrast	—	1.927	0.170	0.032
	—	*I–J*	−2.622	—	—	—

Strength	T0	Contrast	—	0.074	0.786	0.001
	—	*I–J*	−0.212	—	—	—
	T1	Contrast	—	0.536	0.467	0.009
	—	*I–J*	0.696	—	—	—

*Note*: *p*  < 0.05 was considered significant. *I*, control group and *J*, experimental group. Each *F*-tests the simple effects of group within each level combination of the other effects shown. These tests are based on the linearly independent pairwise comparisons among the estimated marginal means. Based on estimated marginal means: *⁣*^*∗*^ The mean difference is significant at the 0.05 level. Adjustment for multiple comparisons: Bonferroni.

**Table 4:** Average value of EEG data, standard deviation, paired sample *t*-test.

Brain Region	Band	T0-Mean (SD)	T1-Mean (SD)	*T*	*p*
Frontal lobe	Theta	10.611	7.039	3.442	0.002*⁣*^*∗*^
	Alpha1	8.440	12.162	−2.139	0.041*⁣*^*∗*^
	Alpha2	3.254	5.830	−3.159	0.004*⁣*^*∗*^
	Beta1	1.172	5.101	−3.082	0.004*⁣*^*∗*^
	Beta2	2.923	4.979	−2.004	0.054
	Gamma	−1.281	0.786	−2.214	0.035*⁣*^*∗*^

M1	Theta	7.517	8.578	−1.606	0.119
	Alpha1	4.521	11.861	−6.493	<0.001*⁣*^*∗*^
	Alpha2	1.327	5.166	−2.934	0.006*⁣*^*∗*^
	Beta1	1.320	3.972	−2.869	0.008*⁣*^*∗*^
	Beta2	1.691	4.037	−2.055	0.049*⁣*^*∗*^
	Gamma	−1.344	−0.123	−1.114	0.274

Parietal lobe	Theta	7.817	7.411	0.470	0.642
	Alpha1	7.012	12.930	−5.315	<0.001*⁣*^*∗*^
	Alpha2	5.546	11.301	−4.120	<0.001*⁣*^*∗*^
	Beta1	3.049	4.812	−2.159	0.039*⁣*^*∗*^
	Beta2	2.584	1.470	1.026	0.314
	Gamma	−1.456	−0.402	−1.125	0.270

Occipital lobe	Theta	12.300	10.459	2.547	0.016*⁣*^*∗*^
	Alpha1	11.824	17.545	−6.332	<0.001*⁣*^*∗*^
	Alpha2	8.122	11.618	−2.581	0.015*⁣*^*∗*^
	Beta1	3.948	7.442	−3.511	0.001*⁣*^*∗*^
	Beta2	5.454	4.843	0.520	0.607
	Gamma	3.508	6.362	−2.197	0.036*⁣*^*∗*^

*Note:⁣* ^*∗*^ *p*  < 0.05 was considered significant. We selected the raw absolute power from the electrodes of interest corresponding to the delta (δ), theta (θ), alpha (α), and beta (β) bands in the affected side area for statistical analysis.

## Data Availability

The data that support the findings of this study are available from the corresponding author upon reasonable request.
